# Effect of nano silver fluoride pre-treatment on the microtensile bond strength of a universal adhesive to dentin: an in vitro study

**DOI:** 10.2340/biid.v13.45648

**Published:** 2026-03-31

**Authors:** Larissa Alexsandra dos Santos Silva, Anna Caroline Lima dos Santos, Luis Felipe Espíndola-Castro, Ana Luísa Cassiano Alves, Márcia de Almeida Durão, Maria Clara Müller de Andrade, André Galembeck, Gabriela Queiroz de Melo Monteiro

**Affiliations:** a Faculty of Dentistry, University of Pernambuco, Recife-PE, Brazil; b Dental School, Federal University of Pernambuco, Recife-PE, Brazil; c Dental School, Faculty of Dentistry of Recife, Recife-PE, Brazil; d Department of Fundamental Chemistry, Federal University of Pernambuco, Recife-PE, Brazil

**Keywords:** silver nanoparticles, dentin adhesion, universal adhesive

## Abstract

**Objective:**

The study aims to evaluate the effect of Nano Silver Fluoride (NSF) pre-treatment at two concentrations on the microtensile bond strength (µTBS) of a universal adhesive to dentin.

**Methods:**

Flat deep coronal dentin surfaces from sound human molars were treated with NSF at 600 or 1500 ppm, or left untreated (control). A universal adhesive (Single Bond Universal; 3M ESPE/now Solventum) was applied in self-etch (SE) mode, followed by placement of a bulk-fill resin composite (Filtek Bulk Fill; 3M ESPE / now Solventum). Specimens were sectioned to obtain sticks (~1 mm^2^) and tested for µTBS using a universal testing machine (500 N load cell; 5 mm/min crosshead speed), considering the tooth as the experimental unit (*n* = 8). Group comparisons were performed using Welch’s one-way Analysis of Variance (ANOVA). Weibull analysis was conducted on stick-level data (*n* = 56 per group) to assess bond strength reliability. Failure modes were evaluated under stereomicroscopy, and dentin morphology and silver nanoparticle distribution were qualitatively assessed using Scanning Electron Microscopy (SEM).

**Results:**

No statistically significant differences were detected among groups (*p* = 0.074). Mean µTBS values were 33.0 ± 10.2 MPa (control), 35.6 ± 8.7 MPa (NSF 600), and 21.4 ± 11.7 MPa (NSF 1500). Effect size analysis indicated a large effect associated with NSF concentration. Weibull analysis showed lower characteristic strength and Weibull modulus for the NSF 1500 group, indicating reduced bond reliability. Adhesive failures predominated in all groups.

**Conclusion:**

Although no statistically significant differences were detected, higher NSF concentration (1500 ppm) was associated with lower bond strength reliability, whereas the lower concentration (600 ppm) maintained favorable bonding performance. Greater accumulation of silver nanoparticles on the dentin surface may interfere with resin infiltration and hybrid layer formation.

**KEY MESSAGES:**

1. The concentration of NSF pre-treatment may influence the adhesive-dentin bond, with higher concentrations (1500 ppm) associated with lower bond strength reliability, although no statistically significant differences were detected.
2. A higher accumulation of silver nanoparticles on dentin surfaces may interfere with adhesive infiltration and compromise the bonding performance of a universal adhesive.
3. NSF at 600 ppm positively influenced universal adhesive performance, preserving the adhesive interface integrity.

## Introduction

Dental caries is a biofilm-mediated and sugar-driven disease that leads to progressive demineralization of dental hard tissues due to the metabolic activity of bacteria, particularly *Streptococcus mutans* [1, 2]. In addition to the mechanical removal of carious tissue, resin composites remain the restorative material of choice for the rehabilitation of the affected tooth as they combine aesthetics with satisfactory mechanical performance. However, their clinical longevity strongly depends on the quality and durability of the adhesive interface. The formation of secondary caries along this interface remains one of the main causes of restoration failure and replacement [3, 4].

Secondary caries is a multifactorial process that integrates the mechanisms of primary caries formation with pathological demineralization along the restoration margin [5, 6]. To address this, various preventive strategies have been proposed to arrest lesion progression, preserve affected dentin, and enhance antibacterial protection at the tooth-restoration interface. Among these approaches, the incorporation of antibacterial agents into dental materials has been explored to limit bacterial invasion and biofilm formation beneath restorations. However, these additives may compromise the mechanical and adhesive properties of the dental material and increase production costs [7, 8].

Silver diamine fluoride (SDF) has long been recognized for its potent anticariogenic effect, combining the antimicrobial action of silver ions with the remineralizing capacity of fluoride [9–11]. Its use as a cariostatic pre-treatment prior to restorative procedures has been proposed to reduce the risk of secondary caries [12]. Nonetheless, several studies have reported that SDF pre-treatment may impair the bond strength of adhesives to dentin, regardless of whether self-etch (SE) or etch-and-rinse (ER) protocols are used [13, 14]. Moreover, the undesirable dark staining of tooth structure caused by SDF limits its acceptance in aesthetic areas [15]. These drawbacks have stimulated the search for alternatives that retain anticariogenic efficacy without compromising appearance or adhesion.

The development of nanotechnology-based formulations in dentistry has led to the introduction of Nano Silver Fluoride (NSF), an innovative cariostatic agent designed to achieve SDF-like antibacterial activity without inducing tooth discolouration [16, 17]. NSF combines silver nanoparticles (AgNPs), chitosan, and fluoride, showing comparable caries-preventive and antibacterial properties to SDF while demonstrating low cytotoxicity at 600 and 1500 ppm concentrations [18]. The antimicrobial mechanism of AgNPs is attributed to their high surface-to-volume ratio, which allows effective penetration through bacterial cell walls, while fluoride and chitosan enhance both antibacterial and remineralizing effects [19, 20]. Additionally, the penetration of AgNPs into dentinal tubules may confer long-lasting cariostatic activity. Previous studies have shown that silver nanoparticles incorporation into adhesives can reduce collagen degradation and enhance bond durability [21]. Nevertheless, the effect of NSF pre-treatment on adhesive bonding to dentin remains poorly understood.

Bond strength is a key parameter for assessing the clinical durability of resin composite restorations. Two main bonding strategies – ER and SE – are available, each presenting specific advantages [22]. Although ER adhesives are still considered the gold standard, universal adhesives have gained popularity due to their versatility and simplified application [23]. Therefore, the present study aimed to evaluate the microtensile bond strength (µTBS) of a universal adhesive to dentin pre-treated with NSF at 600 ppm and 1500 ppm concentrations.

The null hypotheses tested were: (1) NSF pre-treatment does not influence the dentin bond strength; and (2) there is no difference between the two NSF concentrations.

## Materials and methods

All methodological procedures followed the recommendations of the Academy of Dental Materials for *in vitro* testing of bonding effectiveness according to the µTBS guidelines proposed by Armstrong et al. [24].

### Sample size

For statistical purposes, the tooth was considered the experimental unit, whereas the beams obtained from each tooth were treated as subsamples. The sample size was calculated based on data from Guarda et al., using the OpenEpi (Open Source Epidemiological Statistics for Public Health, available at https://www.openepi.com/Menu/OE_Menu.htm) [25]. The calculation considered the mean and standard deviation of the µTBS values reported in that study: 32.16 ± 6.10 MPa for the control group and 44.57 ± 6.82 MPa for the experimental group. A significance level of 5% (α = 0.05) and a statistical power of 90% were adopted. The analysis indicated a minimum of six teeth per group; however, to increase the robustness of results, eight teeth per group were used, totaling 24 sound human molars.

### Specimen preparation

Twenty-four sound human molars, extracted for therapeutic reasons to be free of caries or restorations, were collected following ethical approval by the Research Ethics Committee Involving Human Subjects of the University of Pernambuco (CEP-PROPEGI, protocol number 4.437.214).

Teeth were cleaned and their occlusal enamel was removed using a double-sided diamond disc under water cooling in a low-speed precision cutter (ISOMET 1000, Buehler Ltd., Lake Bluff, IL, USA), thereby exposing deep coronal dentin. To stabilize the specimens during sectioning, the roots were embedded in polyvinyl chloride (PVC) cylinders filled with self-curing acrylic resin. The exposed dentin surfaces were polished with silicon carbide papers of decreasing grit (240, 320, and 600) for 60 seconds each underwater to standardize the smear layer.

The specimens were then randomly assigned to three groups (*n* = 8), according to the dentin pre-treatment protocol:

- CON (Control): No cariostatic agent applied.
- NSF 600: Application of NSF at 600 ppm.
- NSF 1500: Application of NSF at 1500 ppm.

NSF were prepared as follows: an AgNO_3_ aqueous solution (0.11 mol·L^−1^) was added to a chitosan solution (0.01 g.mL^−1^) that had been dissolved in acetic acid (1% v/v). NaBH_4_ (0.66 mol·L^−1^) was added dropwise, resulting in a suspension of silver nanoparticles (AgNPs), which was then supplemented with NaF and stirred until the salt was completely dissolved. The composition and approximate concentrations of the active agents in the cariostatic formulations were chitosan (10,000 ppm), fluoride (5000 ppm), and silver nanoparticles (600 and 1500 ppm). The final pH values of the NSF solutions were 4.08 for NSF 600 ppm and 4.54 for NSF 1500 ppm. The formulations were protected from light exposure and stored at room temperature [26].

For the experimental groups, one drop of NSF was applied to the flat dentin surface using a microbrush under light friction for 1 min as described by Espíndola-Castro et al. [17]. After treatment, all teeth were stored in saline solution at room temperature for 5 days.

### Adhesive procedures

After storage, the dentin surfaces were cleaned with pumice slurry to remove the chitosan pellicle [17] and soft brush for 1 min, rinsed with water, and gently dried with absorbent paper [17].

The universal adhesive (Single Bond Universal; 3M ESPE/ now Solventum, Minnesota, IL, USA) was applied in self-etching mode according to the manufacturer’s instructions: active application with a microbrush for 20 seconds, gentle air-drying for 5 seconds, application of two consecutive layers, and light curing for 20 seconds. A bulk-fill resin composite (Filtek Bulk Fill; 3M ESPE/ now Solventum, Minnesota, IL, USA) was inserted in a single 4 mm increment, forming a restoration block and light-cured for 40 s on the occlusal surface and 5 seconds on each lateral surface (Table 1).

**Table 1:** Materials and application procedures used in the adhesive protocol.

Material LOT	Manufacturer	Composition*	Application mode
Single Bond Universal 2101100955	3M ESPE, Now Solventum (St. Paul, MN, USA)	HEMA, MDP, dimethacrylates, polyalkenoic acid, filler particles, ethanol, water, CQ, silane.	Applied in self-etching mode with a microbrush under friction for 20 s; gentle air-drying for 5 s; two consecutive layers applied; light curing for 20 s (20 J/cm^2^).
Filtek Bulk Fill NC44422	3M ESPE, Now Solventum (St. Paul, MN, USA)	Filler: (% wt.): 76.5, YbF3, zirconium, silica Matrix: Bis‐GMA, Bis‐EMA, UDMA Color shade: A2	Inserted in a single 4 mm increment, light curing for 40 s (occlusally – 40 J/cm^2^) + 5 s per side.
NSF 600 ppm	(Cetene, Pernambuco, Brazil)	Chitosan (10,000 ppm), fluoride (5000 ppm), and silver nanoparticles (600 ppm)	One drop of NSF was applied to the flat dentin surface using a microbrush under light friction for 1 min
NSF 1500 ppm	(Cetene, Pernambuco, Brazil)	Chitosan (10,000 ppm), fluoride (5000 ppm), and silver nanoparticles (1500 ppm)	

* HEMA: (hydroxyethyl) methacrylate; MDP: methacryloyloxydecyl dihydrogen phosphate; CQ: camphorquinone; Bis-GMA: Bisphenol A glycidylmethacrylate; Bis-EMA: Bisphenol A ethoxylated dimethacrylate; UDMA: urethane dimethacrylate.

Light curing was performed using a Light Emitting Diode (LED) unit (Valo Grand Cordless, Ultradent, South Jordan, UT, USA) operated in standard mode, with a manufacturer-reported irradiance of approximately 1000 mW/cm^2^, corresponding to energy densities of 20 and 40 J/cm^2^. Light exposure was standardized for all specimens. The restored teeth were stored in physiological saline solution (0.9% NaCl) at 37ºC for 7 days before testing, following a protocol previously described in the literature [27].

All storage periods were applied equally to all experimental groups and were intended for short-term stabilization and logistical standardization, rather than simulation of long-term aging.

### Microtensile bond strength

Each restored tooth was sectioned with a low-speed water-cooled diamond saw (ISOMET 1000, Buehler Ltd., Lake Bluff, IL, USA) to obtain bar-shaped specimens (~1 mm^2^ cross-section). From each tooth, seven sticks were randomly selected, totaling 56 sticks per group. The adhesive interface area was measured using a digital caliper (accuracy ± 0.01 mm; Mitutoyo, Suzano, SP, Brazil). Each stick was attached to a microtensile testing jig using cyanoacrylate gel and self-curing acrylic resin (VIPI FLASH/VIPI, São Paulo, Brazil) ensuring that the bond interface was exposed. Testing was carried out in a universal testing machine equipped with a 500 N load cell, at a crosshead speed of 5 mm/min until failure. The µTBS in (MPa) was calculated using the formula:


$$m\text{TBS}=\frac{Fx9.8}{A}$$

where *F* is the load at fracture (kgf), *A* is the cross-sectional area (mm²), and 9.8 is the gravitational conversion factor from kgf/mm² to MPa.

### Failure mode analysis

Failure modes were classified under a stereomicroscope (40×) as adhesive, cohesive in dentin, cohesive in resin composite, or mixed, based on the presence or absence of residual resin on the dentin surface.

### Scanning electron microscopy

Representative specimens (*n* = 2 per group) from each group were analyzed using scanning electron microscopy (SEM, Tescan Mira 3, Czech Republic), operated at an accelerating voltage of 5 kV. Samples were mounted on aluminum stubs and carbon-coated. Secondary Electron (SEM/SE) images were used to evaluate dentin surface topography, whereas Basckscattered Electron (SEM/BSE) images were used to qualitatively assess the distribution of silver nanoparticles within the dentin, which appeared as brighter due to their higher atomic number.

### Statistical analysis

Data were initially organized in Microsoft Excel (Microsoft Corp., Redmond, WA, USA). Normality of the data distribution was assessed using the Shapiro–Wilk test. Group comparisons were performed considering the tooth as the experimental unit using Welch’s one-way Analysis of Variance, as a robust approach for small sample sizes. Effect size estimates were calculated to assess the magnitude of the differences among groups.

Weibull analysis was performed using a two-parameter Weibull distribution on µTBS values obtained from individual sticks (*n* = 56 per group). The Weibull modulus (m) and characteristic strength (σ₀) were estimated using linear regression of Weibull plots based on median ranks. Stick-level data were used exclusively for Weibull analysis and were not considered independent observations for inferential statistical testing.

Statistical analyses were performed using jamovi software (version 2.6.44; The jamovi project) and Microsoft Excel. The significance level was set at *p* < 0.05.

## Results

### Microtensile bond strength

The data showed borderline normality (Shapiro–Wilk test, *p* = 0.05). Welch’s one-way ANOVA revealed no statistically significant differences among groups (*n* = 8 per group; *F*(2,13.9) = 3.16, *p* = 0.074). Mean µTBS values were 33.0 ±10.2 (control), 35.6 ± 8.7 (NSF 600), and 21.4 ± 11.7 (NSF 1500), as shown in Table 2. Effect size estimates indicated a large effect associated with NSF concentration (η² = 0.31; ω² = 0.20; Cohen’s *f* = 0.51).

**Table 2:** Mean microtensile bond strength (µTBS) values (MPa ± SD; *n* = 8 teeth per group) and Weibull parameters (*m* and σ₀; *n* = 56 sticks per group) for the experimental groups.

Group	*n* (teeth/sticks)	Mean µTBS (MPa ± SD)	Weibull modulus- m	Characteristic strength – σ₀ (MPa)
CON	8/ 56	33 ± 10.2	1.47	38.52
NSF600	8/ 56	35.6 ± 8.7	1.90	40.70
NSF1500	8/ 56	21.4 ± 11.7	1.29	22.79
***P* value** ^**1**^		**0.074**		

1 Welch’s one-way ANOVA; *p* value refers to mean TBS comparison only. Weibull parameters are descriptive reliability estimates. TBS: tensile bond strength.

Weibull analysis showed that the NSF 1500 group presented lower characteristic strength (σ₀ = 22.79 MPa) and lower Weibull modulus (*m* = 1.29) compared with the control (σ₀ = 38.52 MPa; *m* = 1.47) and NSF 600 groups (σ₀ = 40.70 MPa; *m* = 1.90), indicating reduced bond strength and lower reliability at the higher NSF concentration.

### Failure mode analysis

**Figure 1:**
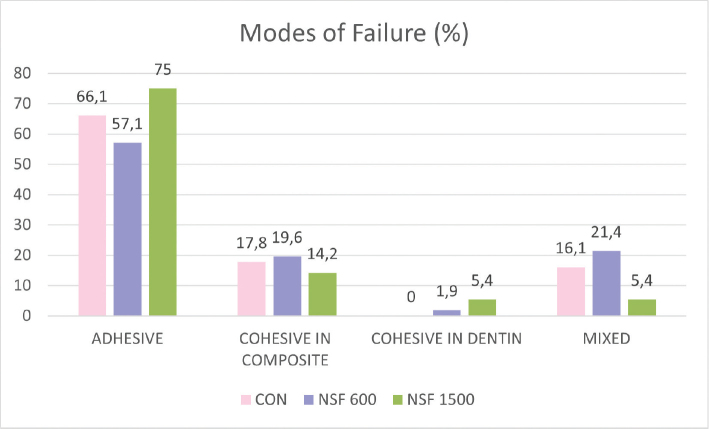
Distribution of failure modes (%) for each experimental group.

The distribution of failure modes is shown in Figure 1. Adhesive failures predominated in all groups (≥ 57%), with the highest percentage observed in the NSF 1500 (75%). Mixed failures were less frequent in the NSF 1500 group, while cohesive failures in composite and dentin occurred at low and comparable frequencies across groups.

### Micromorphological analysis

**Figure 2:**
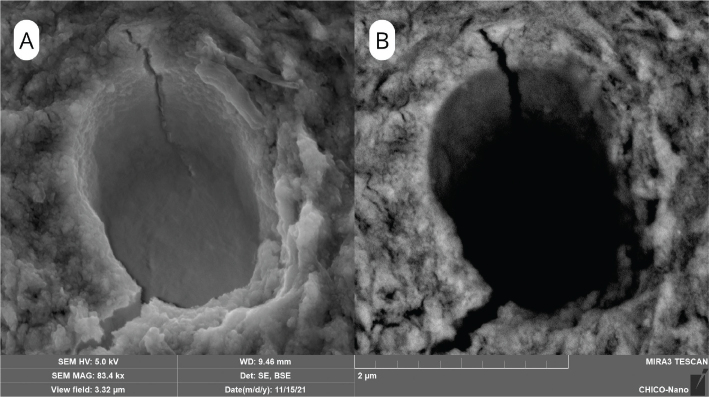
SEM image of dentin in the control group. (A) SEM/SE image showing clean dentinal tubules without deposits. (B) SEM/BSE image confirming absence of silver nanoparticles.

**Figure 3:**
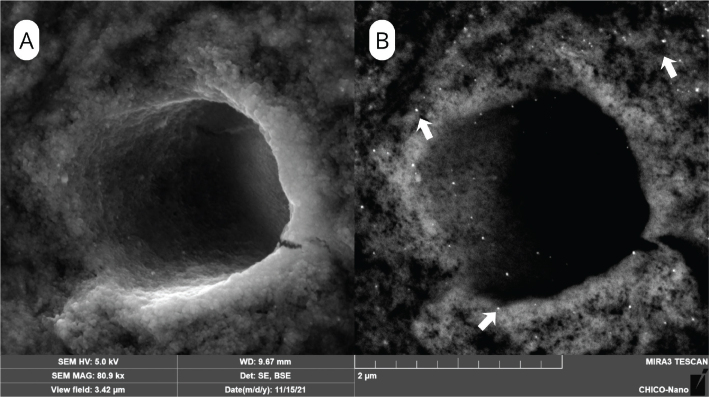
SEM images of dentin treated with Nano Silver Fluoride (NSF) at 600ppm. (A) SEM/SE image highlighting the surface topography of dentinal tubule; (B) SEM/BSE image showing discrete silver nanoparticle deposits (arrows) within the peritubular and intertubular dentin..

**Figure 4:**
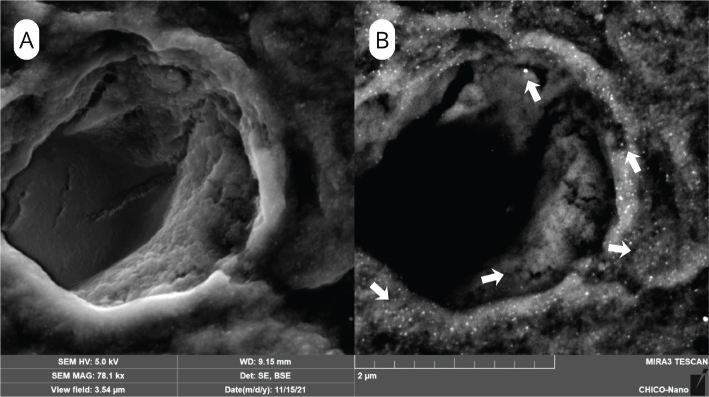
SEM images of dentin treated with Nano Silver Fluoride (NSF) at 1500ppm. A) SEM/SE image illustrating the dentin surface morphology; (B) SEM/BSE image showing intense and widespread silver nanoparticle deposition (arrows) across dentin surface..

SEM surface analyses revealed clear morphological differences among groups. The SEM surface characteristics of the control group are presented in Figure 2, which shows open dentinal tubules with no evidence of silver particle deposition. After NSF treatment, SEM/BSE images demonstrated bright deposits of silver nanoparticles concentrated along peritubular and intertubular dentin, more evident at 600 ppm (Figure 3) than at 1500 ppm (Figure 4).

## Discussion

The results of this study did not show sufficient statistical evidence to reject the null hypotheses, indicating that NSF pre-treatment did not affect dentin bond strength and that there was no difference between the NSF concentrations evaluated. However, the findings indicate a concentration-dependent tendency of NSF on dentin bonding. Although statistical significance was not achieved, the combination of lower mean bond strength values, greater variability, and reduced bond reliability observed at the higher NSF concentration points to a tendency toward impaired adhesive performance. This tendency may reflect a potential adverse effect of higher silver nanoparticle concentrations on the integrity of the adhesive interface, particularly in deep dentin substrates.

Deep coronal dentin was selected in this study because dentin depth is a critical factor influencing adhesive performance and represents a more clinically challenging substrate. Compared with superficial dentin, deep dentin exhibits a higher density and larger diameter of dentinal tubules, reduced intertubular dentin, and increased intrinsic moisture, all of which negatively affect resin infiltration and hybrid layer formation [28, 29].

Previous studies have demonstrated that dentin depth significantly influences bond strength outcomes, with lower values generally observed in deep dentin. However, Gokce et al., reported that Single Bond Universal maintained superior adhesive performance compared with other universal adhesives, regardless of dentin depth. This behavior suggests that this adhesive can achieve reliable bonding even under the unfavorable conditions typically found in deep dentin, reinforcing its clinical applicability in deep cavities [30].

The storage protocol adopted in the present study was designed to allow initial stabilization of the dentin-adhesive interface and completion of polymerization reactions before mechanical testing [27, 31]. The 7-day storage period minimizes the influence of immediate post-polymerization effects and facilitates comparison with previous bond strength studies. Importantly, all storage periods were applied equally to all experimental groups and were intended for short-term stabilization and logistical standardization, rather than simulation of long-term aging.

Previous research using SDF as a cariostatic pre-treatment has produced contradictory results regarding its impact on bond strength. Some studies have shown that SDF can decrease adhesion, depending on the adhesive, whereas others reported no detrimental effect [13, 32, 33]. Although SDF effectively arrests caries, its pronounced staining of dental tissues remains a major aesthetic concern, limiting its use in visible areas [34, 35]. Additionally, potential cytotoxic effects related to the high concentrations of silver and fluoride ions raise safety concerns for applications near the dental pulp [36].

In this context, NSF emerges as a promising alternative, providing strong antibacterial and remineralizing effects without tooth discoloration [17]. Recent evidence has confirmed that NSF 600 and 1500 ppm presents no transdentinal cytotoxicity and may thus be safely applied on dentin surfaces [18]. Furthermore, Das et al. observed that dentin pre-treated with NSF improved the bond strength of conventional and resin-modified glass ionomer cements, although the concentration of AgNPs was not specified [37].

Siqueira et al. showed that the application of 38% diamine resulted in significantly higher bond strength values in the SE approach for both adhesives compared with 12% diamine, while no significant differences were observed between adhesive strategies overall [38]. In contrast, previous studies have reported that silver nanoparticles incorporated into dental adhesives may positively influence bond strength particularly when combined with ER and applied prior to acid etching [27]. In the present study, however, the SE mode was intentionally selected due to frequent clinical use with universal adhesives and its reduced technique sensitivity, especially when bonding to deep coronal dentin [39].

SEM analysis supported this interpretation, revealing a denser accumulation of silver nanoparticles in the NSF 1500 group, mainly along the peritubular and intratubular dentin. Taken together, SEM observations indicated that greater nanoparticle density at higher NSF concentration was associated with lower bond strength values, suggesting possible interference with adhesive infiltration. According to Zecin-Deren et al., adequate penetration and polymerization of the adhesive resin within the demineralized collagen network are essential for achieving strong and durable bonds [40]. When nanoparticles accumulate on the dentin surface, they may obstruct adhesive monomer infiltration and modify the permeability and surface characteristics, thereby reducing bond reliability.

In contrast, the better performance of NSF 600 may be related to a more uniform and superficial distribution of silver nanoparticles, allowing adequate adhesive diffusion while still providing antimicrobial protection. Lutgen et al. demonstrated that the removal of excess silver precipitates improved bonding performance in SDF-treated dentin [13]. In the present study, although no immediate rinsing step was performed after NSF application, specimens were stored in physiological saline solution for 5 days and subsequently subjected to standardized prophylaxis prior to adhesive procedures. Despite these measures, the higher NSF concentration may have resulted in greater residual nanoparticle accumulation within the dentin substrate, contributing to reduced bond reliability. Future studies should investigate modified application protocols, including immediate rinsing or controlled surface conditioning, to optimize adhesive performance following NSF pre-treatment.

Clinically, these results highlight the importance of the concentration of NSF used for dentin pre-treatment. Lower concentrations (600 ppm) appear to preserve favorable bonding performance while offering anticariogenic benefits [41]. From a restorative perspective, NSF at 600 ppm may represent a feasible adjunct in minimally invasive dentistry, where both bacterial control and bonding integrity are essential.

However, this study has limitations. As an *in vitro* experiment, it does not fully replicate the complex intraoral environment, including temperature fluctuations, pH variations, and masticatory stresses. No aging protocols were performed, preventing the assessment of long-term bonding behavior. Additionally, only one adhesive and one bonding strategy were evaluated, limiting the generalizability of the findings. Another limitation is the use of sound dentin, despite the clinical relevance of NSF in caries management. Sound dentin was intentionally selected to ensure substrate standardization and to isolate the effect of NSF concentration on adhesive bonding. Therefore, extrapolation of these findings to caries-affected dentin should be made with caution. Future studies should investigate aged specimens, caries-affected dentin, different adhesive formulations, and modified NSF application protocols to better reflect clinical conditions.

## Conclusions

Within the limitations of this *in vitro* study, NSF pre-treatment at 1500 ppm tended to reduce dentin bond strength reliability, whereas 600 ppm maintained favorable bonding performance. Although no statistically significant differences were observed, effect size and Weibull analyses suggest that NSF concentration plays a potentially relevant role in adhesive-dentin interaction.

Taken together, these findings suggest a concentration-dependent tendency rather than a statistically conclusive effect of NSF on dentin bond strength. Further research, including long-term aging protocols and studies using caries-affected dentin, is needed to clarify the clinical relevance of these observations.

## Data Availability

Not applicable.
